# Adaptation and Evaluation of Myfood24-Germany: A Web-Based Self-Administered 24-h Dietary Recall for the German Adult Population

**DOI:** 10.3390/nu12010160

**Published:** 2020-01-06

**Authors:** Stefanie A. J. Koch, Johanna Conrad, Linda Hierath, Neil Hancock, Sarah Beer, Janet E. Cade, Ute Nöthlings

**Affiliations:** 1 Nutritional Epidemiology, Department of Nutrition and Food Sciences, University of Bonn, 53115 Bonn, North Rhine-Westphalia, Germany; jconrad@uni-bonn.de (J.C.); lhierath@uni-bonn.de (L.H.); noethlings@uni-bonn.de (U.N.); 2 Nutritional Epidemiology Group, School of Food Science and Nutrition, University of Leeds, Leeds LS2 9JT, UK; n.hancock@leeds.ac.uk (N.H.); J.E.Cade@leeds.ac.uk (J.E.C.); 3 Dietary Assessment Ltd., Nexus, University of Leeds, Leeds LS2 3AA, UK; S.L.Beer@leeds.ac.uk

**Keywords:** dietary assessment, web-based 24-h dietary recall, myfood24, technology, epidemiological studies

## Abstract

Our aim was to develop and evaluate a German adaptation of myfood24, a fully automated, web-based 24-h dietary recall (24HDR). To complete a self-administered 24HDR with myfood24, users have to search and enter consumed foods within the underlying database by a free text search. The adaptation process thus mainly consisted of the development of an appropriate food database. myfood24-Germany was evaluated in 92 adults aged 17–78 years (study 1). Participants completed four non-consecutive 24HDRs and answered an evaluation questionnaire after the final recall. The System Usability Scale Score (SUS Score, 0–100) was calculated. Users’ search behavior was examined with screen recordings in 15 adults aged 20–60 years (study 2). Participants had to enter three sample meals presented as food packaging or pictures. The final database included 11,501 food items (7203 generic and 4298 branded items) with up to 131 nutrients. In study 1, the median completion time for a 24HDR was 15 min. The median SUS score of 78 indicated good usability. The majority of participants considered the overall user-friendliness as good (46%) or very good (21%), and 75% were willing to use myfood24-Germany regularly. Both studies showed that finding and choosing an appropriate item within the database was a major challenge. A German version of myfood24 was successfully developed. The user evaluation indicated a short completion time, good usability and acceptability of the tool, and confirmed its feasibility for repeated short-term application.

## 1. Introduction

The repeated application of short-term dietary assessment instruments such as 24-h dietary recalls (24HDRs) has been recommended to accurately estimate the usual dietary intake in large-scale studies [1,2,3]. The administration of multiple interviewers led 24HDRs in large populations is costly and time-consuming. Web-based self-administered 24HDRs are promising to facilitate dietary assessment in epidemiological studies, reducing the costs and time effort that have been associated with repeated administrations of traditional instruments [4]. An essential feature of web-based tools is that they can be adapted to other databases, which allows the administration of the same tool across countries [5]. With respect to the standardization of dietary assessment across countries, the adaptation of existing tools should be preferred to the development of new instruments. The main task within the adaptation process for most systems is the development and formatting of a country-specific food composition database. Due to the self-administered nature of online tools, a more user-friendly database is required as compared to the interviewer-led counterparts [6]. Participants have to identify and select an appropriate item for a consumed food directly from the underlying database. Therefore, the underlying database should include a wide range of well-defined common food descriptions, while meeting requirements for use in scientific research, i.e., providing verifiably reliable nutrient data [6].

Myfood24 is a fully automated online dietary assessment tool, which was first developed for use in the UK population [7]. It can be used as a self- or interviewer-administered 24HDR or food record (FR) and has been shown to be valid and suitable for dietary assessment in adolescents and adults in the UK [7,8,9,10]. Its web-interface for participants is designed to lead the user through the 24HDR [7,11]. The main features are: (1) an optional quick list to list everything consumed in a brainstorming approach (once the list is completed, the search function works its way through this list); (2) a recipe builder, where users can log ingredient combinations; (3) a list of recently used items for quick entry of repeated foods/drinks; (4) a help function with detailed help texts and videos; and (5) search filters that can be used to filter search results by category or brand. Other features are portion size options, images, and pop-up windows for commonly forgotten foods (e.g., milk in coffee) and in case of presumably unrealistic portion sizes. Further, myfood24 features an additional web-interface for researchers, where different research projects can be created, customized, and administered by adding project-specific texts and logos, tailored invitations, and reminder emails. Detailed information on the development process and other features of myfood24 is given elsewhere [7].

To our knowledge, so far there is no web-based 24HDR available for use in the German population. Therefore, we aimed to develop a German adaptation of myfood24 and evaluate its feasibility and usability in a German study sample.

## 2. Materials and Methods 

### 2.1. Adapting myfood24 for Use in Germany

The adaptation process of myfood24 for use in German populations included three major steps: (1) the compilation of a German food composition database suitable for self-administered use; (2) its formatting according to the predefined myfood24 database structure; and (3) the translation of all implemented text modules to the German language. The compilation process was conducted by a team of experienced researchers in nutritional epidemiology with knowledge of German consumption habits. All translations were double-checked.

The myfood24-Germany database was built from two German food composition databases—the German Food Code and Nutrient Data Base (Bundeslebensmittelschlüssel (BLS) version 3.02) and the in-house database of the Dortmund Nutritional and Anthropometric Longitudinally Designed (DONALD) study LEBTAB [12,13,14]. The BLS has been developed as a standard instrument for assessing energy and nutrient intakes of epidemiological studies and nutritional surveys in Germany and is freely available for scientific research projects [12,15]. It contains around 15,000 generic food, drink, and recipe items assigned to 131 nutrients. In order to make the BLS items suitable for use in a self-administered online tool, an editing process with the following steps was conducted: (1) removing items that are likely not consumed due to their nature or processing (e.g., bovine blood or kitchen waste); (2) excluding combined food items when their single components were quantifiable (e.g., bread with butter and cream cheese); (3) excluding duplications that result from the hierarchical structure of the BLS; (4) removing dietary supplements and diabetic products; (5) merging items similar by definition and in nutrient composition (<10% deviation in nutrient values); and (6) changing technical item descriptions to more comprehensible ones (e.g., mixed milk products to dairy drinks). For selected food items, representative data of the German National Nutrition Survey II (NVS II) were used to determine whether a specific BLS food or drink item was consumed, followed by a decision on whether to include this item to the myfood24-Germany database or not [16]. Moreover, a few branded food items from the NVS II food list were added to the database that were already matched to a generic BLS food code. 

As the BLS includes generic food items only, selected branded food items from LEBTAB [14] were added to the database of myfood24-Germany. LEBTAB currently contains about 13,000 generic and branded food items with corresponding energy and nutrient contents (75 nutrients) and is continuously updated [14,17]. Data on nutrient content of branded products is derived from a recipe simulation process based on the back of pack data on ingredients and nutrient information [14]. For myfood24-Germany, only recently added branded food products that were still available on the market were selected. Like the BLS items, LEBTAB descriptions of food items were edited. 

To be able to use the myfood24 features, further formatting steps were necessary. myfood24 includes prompts for often forgotten food items. Therefore, common accompaniments to foods were added to certain items (e.g., sugar to coffee). Second, a number of synonyms (e.g., dialect variations) and potential misspellings (e.g., “spagetti” for “spaghetti”) were added. Third, filters were formatted by assigning information on type (branded vs. generic) and on food category (e.g., fresh fruit vs. fruit juice) to each food item. Finally, information on average portion sizes and household measures for myfood24-Germany were obtained from two scientific German publications. A nutrition table published by Wahrburg and Egert [18] and the MONICA (Multinational MONItoring of trends and determinants in CArdiovascular disease) Mengenliste, a reference book for average portion sizes published by the Federal Office of Agriculture and Food [19]. For very few missing cases, information from a commercially published nutrition table was used [20]. Pack sizes for branded products were searched on the Internet or were adopted from LEBTAB. Portion images were adopted from the myfood24-UK [5,11,21]. The images were allocated to a wide range of selected food items to support portion size selection. Similar to myfood24-UK, seven portion size images were assigned per food item.

### 2.2. Evaluation of myfood24-Germany (Study 1)

#### 2.2.1. Recruitment and Study Design

The evaluation study of myfood24-Germany was embedded in the validation study of the tool (results on validity will be published separately). Participants were recruited at the campus of the University of Bonn by oral advertisement and flyers as well as in the general German population in and around the city of Bonn by social media and press releases. Eligible subjects had to be ≥18 years old, fluent in German, and had to have regular high-speed Internet access and a valid email address. Further, they had to be stable in bodyweight (i.e., not on a weight-loss diet), free from metabolic diseases, and willing to maintain their current dietary and activity behavior during the time of the study. On a first study visit, individuals were screened for eligibility and informed about the study procedures. During the study, participants were asked to keep a three-day weighed FR and completed four 24HDRs with myfood24-Germany. The first 24HDR was completed in the study center where participants received brief instructions in using myfood24-Germany by study staff. The introduction included a short explanation of the main features (search function, search filters, portion size selection, and recipe builder) for all participants before they started the recall. A researcher was present while participants completed the recall and participants were allowed to ask questions on functionalities and to ask for help when any problems occurred. The following three 24HDRs were completed at home. After the final recall, participants were asked to answer an online evaluation questionnaire.

#### 2.2.2. Assessment of Usability and Acceptability of myfood24-Germany

Data on usability and acceptability of myfood24-Germany was collected by an online evaluation questionnaire including 68 questions on six different categories (usability, technical issues, participant-interface, search for food items and features, time for completion and acceptance, personal comments and suggestions). Usability was assessed by the System Usability Scale (SUS), which is a common method for evaluating digital technologies, conceived by Brooke et al. [22,23,24]. It consists of 10 statements asking the respondent to indicate the level of agreement on a 5-point Likert scale (1 = strongly disagree; 5 = strongly agree). Based on the responses, a score is calculated ranging 0–100, where higher scores indicate better usability. Bangor et al. provided an adjective rating that correlates with a given SUS score [24,25]. In the present study, an appropriate German translation of the SUS developed by SAP usability professionals of a German software corporation (SAP SE; Systems, Applications & Products in Data Processing Societas Europaea) was used [26]. In addition, participants were asked to classify the overall user-friendliness of myfood24-Germany as “bad”, “OK”, “good”, or “very good”.

In order to assess the acceptance of myfood24-Germany, study participants were asked whether they would consider using myfood24 regularly (yes/no) and, if yes, in what time intervals they would use it (once a week, biweekly, once a month, bimonthly, quarterly, 1–2 times/year).

Further, participants were asked about technical details (e.g., commonly used browser and operating system), technical problems (e.g., issues with loading/opening the webpage, technical problems while using myfood24-Germany), their subjective opinion on the user-interface and about the application of the search function, and other features in myfood24 Germany. Finally, participants were able to give free text comments and to make overall suggestions at the end of the questionnaire.

#### 2.2.3. Assessment of Further Covariates

Demographic characteristics and information on lifestyle factors were assessed by an online questionnaire that was filled in by participants in the study center. Bodyweight was measured to the nearest 100 g using a calibrated electronic scale (Kern MPC 250K100M) with participants dressed in underwear only. Standing height was measured to the nearest of 0, 1 cm using a stadiometer (seca 217). Body Mass Index (BMI) was calculated as the bodyweight (kg) divided by the square of the body height (m).

#### 2.2.4. Statistical Analyses

Since only a few variables were normally distributed, descriptive data are presented as medians with their lower- (Q1) and upper-quartile (Q3) for continuous variables or as frequencies and percentages for categorical variables. A backward selection procedure (PROC GLMSELECT in SAS^®^) was used to identify characteristics of participants that were potential predictors for the SUS score or the average completion time for a 24HDR with myfood24-Germany. The following variables were tested: sex (male/female), age (years), BMI (kg/m^2^), education level (no school-leaving qualification/trainee or student/vocational education/college or university degree), nutritional education/profession (“Do you have a food scientific background (e.g., a degree in nutrition/food science, training or education in nutrition,…)?” yes/no), experience in completing 24HDRs (“Before you participated in this study, have you ever done a 24-h dietary recall?” yes/no), experience in using computer (“Do you work with a computer/laptop regularly (>5×/week)?” yes/no), type of diet (“Are you a vegetarian or vegan?” no/yes vegetarian/yes vegan), smoker (yes/no)). The significance level was set at *p* < 0.05. The completion time for a 24HDR was determined by calculating the minutes between the addition of the first and the last item in myfood24-Germany. Of note, participants were free to postpone the completion of their 24HDR and finalize it at a later time point. Therefore, for all 24HDRs completed at home, outliers (>1.5× IQR) in completion time were manually checked by reviewing the automatic myfood24-Germany nutrient data output (*n* = 27). As a result, recalls with time intervals of >60 min between the entry of one item to the next were excluded for analysis of the completion time (*n* = 5).

### 2.3. Evaluation of Participants’ Individual Search Strategy (Study 2)

#### 2.3.1. Recruitment and Study Design

**Figure 1:**
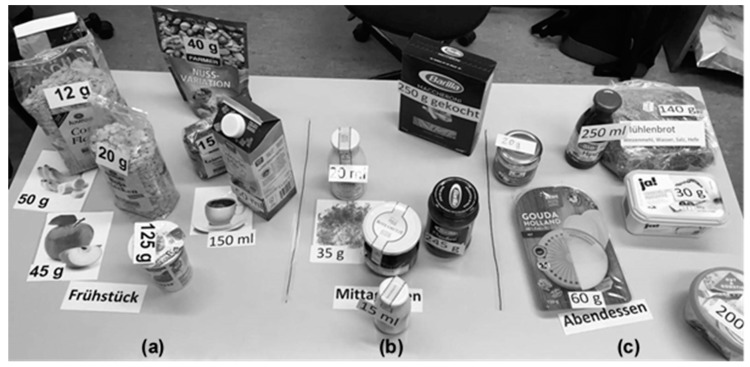
Sample meals presented to study participants. (**a**) Breakfast consisted of *n* = 10 food items (*n* = 3 generic/*n* = 7 branded; *n* = 3 in the database/*n* = 7 not specifically in the database), (**b**) lunch consisted of *n* = 6 food items (*n* = 1 generic/*n* = 5 branded; *n* = 3 in the database/*n* = 3 not specifically in the database), and (**c**) dinner consisted of *n* = 6 food items (*n* = 1 generic/*n* = 5 branded; *n* = 6 not specifically in the database).

To examine users’ search behavior in myfood24-Germany, a convenience sample of students and employees was recruited by oral advertisement and flyers at the University of Bonn (independent of study 1). Subjects had to be ≥18 years old and fluent in German. Nutrition professionals and students of Nutritional Sciences were excluded. Written informed consent from all participants was obtained before enrolment. At the study visit, participants were requested to enter three sample meals (breakfast, lunch, and dinner) in myfood24-Germany (Figure 1). The meal constituents were presented on a table in the study room. Each meal comprised both generic (presented as pictures) and branded items (presented in original packaging) labeled with a hypothetical quantity of consumption. In total, 22 food items were presented of which *n* = 17 were branded products. On purpose, the majority of selected products (*n* = 16) were not specifically available in the myfood24-Germany database so that participants had to find a suitable equivalent (e.g., noodles for “Barilla maccheroni”). Participants received written instructions and had to enter the items without any further help. The entering process was documented with the screen recorder software Apowersoft Free [27]. Demographic characteristics were assessed by a questionnaire.

#### 2.3.2. Statistical Analyses

To investigate users’ search behavior, screen videos were analyzed (analysis 1). First, the number of entered search terms before an item was selected was counted. Second, it was examined overall and stratified by meal, whether participants searched a food item by product name and/or brand name. Finally, it was analyzed how often participants omitted an item (i.e., search terms were entered but no item was chosen) instead of choosing an equivalent product, and how often a food item from the table was forgotten (i.e., participant did not search for a specific item at all). To investigate the impact of individual search behaviors on hypothetical energy and nutrient intakes, mean energy and nutrient intake according to the automatic myfood24-Germany output were compared to the nutrient reference values from the packaging for branded food items or to nutrient information from the BLS for generic products (analysis 2). The mean of absolute individual differences was calculated.

From 18 recruited participants, 15 entered the sample meals following the study protocol (Figure S1). For analysis 1, one participant had to be excluded because the entering process was not recorded due to technical problems. For analysis 2, only nine of 15 entered 24HDRs could be considered because participants who overlooked and forgot some items had to be excluded.

## 3. Results

### 3.1. myfood24-Germany

**Figure 2:**
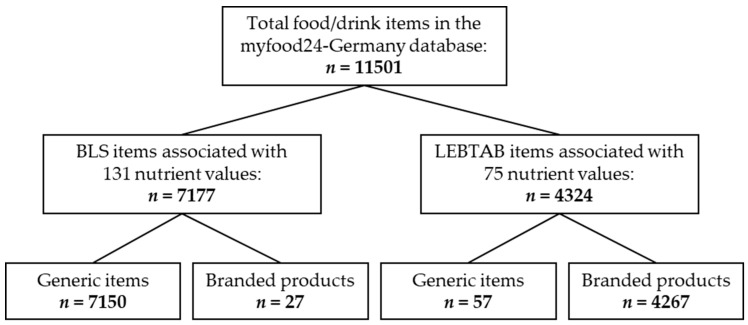
Flow chart of the myfood24-Germany database entries.

A German version of myfood24 including features and functionalities of the original UK version was successfully developed. The current version of the underlying food composition database comprises data from the BLS and LEBTAB (Figure 2). Researchers can choose to use BLS data only (7177 items) or to combine it with data from LEBTAB (4324 items) depending on the study objectives. The combined database contains 11,501 food and drink items with 7203 generic items and 4298 branded products. Items from the BLS and LEBTAB are associated with 131 and 75 nutrient values, respectively. Overall, 359 food portion images from the Young Person’s Food Atlas Secondary publication were allocated to 1230 (11%) of the included food and drink items. The database will be updated at reasonable time intervals.

### 3.2. Evaluation of myfood24-Germany (Study 1)

From 97 study participants, 92 completed the evaluation questionnaire (Table 1). All of these participants completed four 24HDRs with myfood24-Germany, except for one who conducted only three 24HDRs. The majority of participants were female (76%) and had high educational status and experience in using computers. Median age was 30.5 years (Q1–Q3: 25.0–61.5). The age of participants ranged 17–78 years, as one participant turned 18 during the course of the study. A lower number of men than women were recruited with a more limited age range. Median BMI was 23.1 (Q1–Q3: 20.8–24.4) for male and 21.8 (Q1–Q3: 19.6–23.7) for female participants. About 21% of the participants indicated having a nutritional or food scientific background while 11% reported having experience in completing a 24HDR.

**Figure 3:**
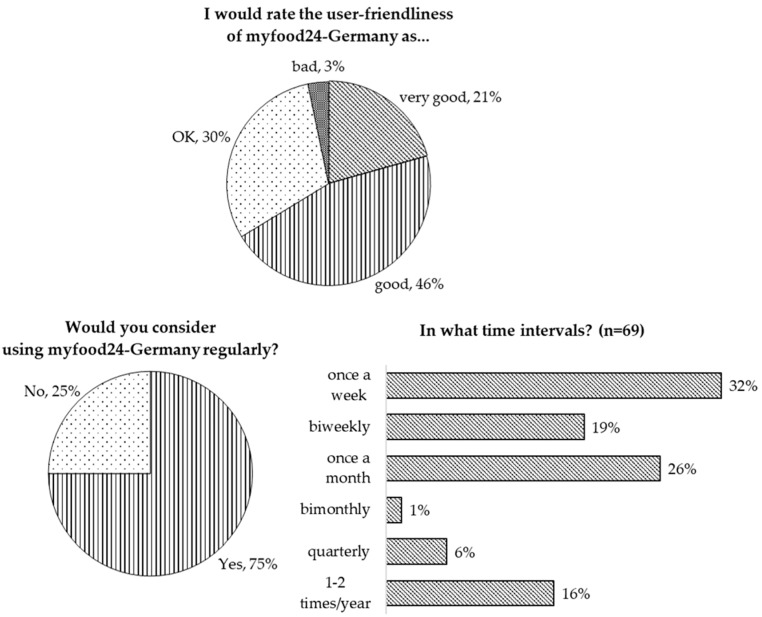
User rating of the overall user-friendliness and acceptance of myfood24-Germany in participants completing an evaluation questionnaire (*n* = 92).

Table 2 shows the SUS scores for myfood24-Germany for the total study sample and stratified by age groups. The median SUS score was good (78, Q1–Q3: 66–84) indicating the decent usability of myfood24-Germany. Among older adults (>65 years), the median SUS score was good (74, Q1–Q3: 70–83), but lower than in the other two age-strata (78, Q1–Q3: 66–85 and 65–85). However, sex was the only characteristic identified as a potential predictor for the SUS score in the examined sample (*p* = 0.04). Median SUS score was lower in women, representing the majority of study participants (median SUS score (Q1–Q3) women vs. men: 75 (65–83) vs. 83 (70–90)). The overall SUS score did not change when nutritionists (*n* = 19) and participants with experience in completing a 24HDR (*n* = 10) were excluded from the calculation (excluded: *n* = 23, median SUS score (Q1–Q3) = 78 (68–83)). In accordance with the good SUS score, the majority of participants considered the overall user-friendliness of myfood24-Germany as good (46%) or very good (21%) (Figure 3). Seventy-five percent agreed that they could imagine using myfood24 regularly, many of them once a week (32%), biweekly (19%), or once a month (26%). Further, only 20% of respondents agreed that keeping one three-day weighed FR was less burdensome than completing four 24HDRs with myfood24-Germany. 

The calculated median time was between the addition of the first and the last food item, and thus the median time it took participants to complete a 24HDR with myfood24-Germany was 15 min (Q1–Q3: 9–23). However, in some cases, >60 min passed from the entry of one food item to the next, suggesting that participants took a break during the entering process. Time requirement increased with age (Table 2). Backward selection analysis showed that age (*p* < 0.0001) was a potential predictor for the individual mean completion time in the examined sample. The completion time did not change after the exclusion of nutritionists and participants with experience in conducting 24HDRs (*n* = 23 excluded, median time for completion in minutes (Q1–Q3) = 15 (8–23)). In concordance with the actual time requirement, the self-reported median completion time was 15 min (Q1–Q3: 10–20).

Ninety percent of participants used a computer or laptop to conduct the 24HDRs and three percent used tablets. Although participants were informed that myfood24 is an online tool, which at the time of study had not been optimized for smartphone application, six participants (7%) reported completing the 24HDRs on a smartphone. All 92 participants agreed that they were able to load the website and 95% agreed that no technical problems occurred during the application. Technical problems described included that food items could not be entered because they have not been displayed properly (*n* = 1), that the correct portion size could not be entered (*n* = 1) and that no confirmation was received after the completion of the recall (*n* = 2).

The majority of participants agreed that the website is easy to navigate (97%) and the layout is appealing (88%). Only three participants stated that they had difficulties with the application of myfood24. The difficulties described by these participants mostly concerned the search for food items. 

The search function in myfood24 was rated as easy to use by 79% of participants and 84% stated that they were able to find food items quickly. Although 71% of participants agreed that they could not find all the foods they consumed, the majority of these participants (74%) agreed that they found appropriate alternatives for the respective food item. Nevertheless, slightly more than half of all participants (53%) stated that they had difficulties in finding an appropriate food item. Mostly, participants described that the database was incomplete, that there should be more basic food items to choose from, better possibilities to enter out-of-home consumption, and the opportunity to add food items that are apparently missing in the database. Further, the alphabetically sorted display and the high number of search results was criticized and a lack of synonyms and (miss)spellings was mentioned. Some participants stated that they would prefer using myfood24-Germany on a smartphone.

The available portion images and portion sizes were found to be helpful by the vast majority of participants (93% and 96%, respectively). Some participants mentioned that creating recipes in the recipe builder was very laborious. Although myfood24-Germany features were briefly explained to participants during the first recall, some participants mentioned that they did not recognize many of the features at all or only after repeated usage. Features in myfood24 used and considered helpful by the users can be seen in Table 3.

### 3.3. Evaluation of Participants’ Individual Search Strategy (Study 2)

Characteristics of participants in study 2 are shown in Table 4. The majority of participants were male (67%), highly educated, and 20 to 60 years old.

#### 3.3.1. Analyses of Individual Search Strategies (Analysis 1)

On average, participants entered two search terms before they selected an item (min 1, max 14) (data not shown). Most participants searched for branded food items by entering the generic descriptions only. Overall, in 21% of the searches for branded items (*n* = 44 of *n* = 238 possible searches given by *n* = 17 products × *n* = 14 participants), participants tried to find the product by further entering the brand name or using the filter option for brands. A sensitivity analysis stratified by meals showed that the entered search terms at the beginning of the entering process included the brand name more often than at the end (breakfast: 31% (30 of 98); lunch: 19% (13 of 70); dinner: 9% (6 of 70); data not shown). When searching for a branded product not identically available in the database, participants either chose a related generic item (69%), another item of the same brand (8%), an equal or similar item from another brand (19%), or they did not choose any item at all (3%). In the remaining cases (1%), the participants forgot to enter the presented food items. When searching for an available branded item (*n* = 2), participants chose the appropriate product in 50% of the cases. For the remaining cases, a related generic item (47%) or a similar product from another brand (3%) was selected.

#### 3.3.2. Comparison of Energy and Nutrient Intakes (Analysis 2)

The comparison of hypothetical mean energy and nutrient intakes derived from the meal entries with reference values is shown in Table 5. Individual contents ranged from 2587 to 3213 kcal for energy, from 77 to 87 g for protein, from 110 to 150 g for fat, and from 310 to 373 g for carbohydrates (data not shown). Mean differences for energy and macronutrients were low (Table 5). The mean of absolute individual differences was 190 kcal for energy, 3 g for protein, 15 g for fat, and 31 g for carbohydrates (data not shown).

## 4. Discussion

A German version of myfood24 has been successfully developed and evaluated with respect to different aspects of usability in a convenience sample of 92 adults. With 15 min completion time, myfood24-Germany was found to be a quick-to-complete, self-administered 24HDR. The SUS score and participants’ willingness to use myfood24-Germany regularly indicated good usability and acceptance in the examined study sample. Further, the majority of the participants judged the repeated use of myfood24-Germany less burdensome than keeping one three-day weighed FR. The majority of participants in our evaluation study agreed that the website of myfood24-Germany was easy to navigate and that they did not have difficulties with the application of the tool. Although most participants of the evaluation study reported that they recognized the available features in myfood24-Germany, they were rarely used according to self-report in study 1 and screen recordings within study 2. Researchers using myfood24-Germany should draw participants’ attention to the available features, as they were found to be helpful when used in study 1.

The SUS score had been developed as a means to measure the overall perceived usability of a system [22]. Although it is not a diagnostic tool, it offers the possibility to compare systems used in the same context [23]. The median SUS score of 78 for myfood24-Germany was comparable to the SUS score of myfood24-UK (median SUS score = 80, *n* = 20) [5] and other web-based dietary assessment instruments, e.g., INTAKE24 (median SUS score = 83, *n* = 20) [28]. Moreover, we found no substantial difference in SUS scores across age groups. In contrast, Carter et al. observed a lower SUS score for myfood24-UK in older adults (median SUS score = 29, *n* = 4 ≥ 65 years old) compared to younger age groups [5]. Further, feasibility testing for ASA24 in two different samples of elderly individuals showed that participants mostly preferred the traditional telephone-administered 24HDR [29] and the SUS score of 57 (SD 12.4, ASA24-Canada-2014) indicated poor usability [30]. The authors presumed that low computer literacy and limited Internet access narrow the ability to conduct self-administered 24HDRs online in older adults [29,30,31]. Our contrasting findings might be explained by the fact that only participants with Internet access were included in the evaluation study, and that the majority of participants had experience in using computers. Ward et al. recently found that technological readiness was not associated with the odds of completing a 24HDR with myfood24-UK in a sample of older adults (*n* = 299 aged 60–85 years) [32]. However, similar to our study population, the examined participants were cognitively healthy, highly educated, and variation across education level was limited. Other age dependent factors (e.g., lack of memory, vision problems) as well as sociodemographic factors might generally affect the ability to conduct self-administered 24HDRs online [29,30,31].

The median completion time of 15 min for myfood24-Germany in the evaluation study was short and comparable to other web-based 24HDRs within every age group [10,33]. For older participants, the completion time was marginally longer. Age was identified as a potential predictor for the individual mean completion time. However, due to the overall small sample size and the sample imbalance between men and women, this result cannot clearly be separated from the impact of sex. Nevertheless, focus group feedback on myfood24-UK also showed that older individuals were willing to spend more time completing a 24HDR online [5]. Kirkpatrick et al. reported that longer completion times of older adults might not only be explained by more application problems but also by higher accuracy in entering items and greater patience [34]. In some cases (*n* = 5), large time gaps between the entry of one food item to the next were observed within study 1. The self-administered 24HDR variant of myfood24-Germany evaluated here enables participants to decide at what time of the day to start and complete the 24HDR and thus offers great flexibility and time saving compared to a traditional interviewer-based 24HDR. The individual completion procedure (e.g., starting the 24HDR in the morning and finishing it when there is time), however, does not always comply with the typical approach of a traditional 24HDR and might affect participants’ memory. The quality of the assessed nutritional data will be determined separately and will give insights on the validity of the self-administered variant of myfood24-Germany. The additional use of the interviewer-administered variant might be beneficial for the inclusion of study participants without Internet access or low computer literacy. However, we cannot draw a conclusion on time requirements or the usability and feasibility of this variant of myfood24-Germany from the present studies.

Some participants (*n* = 6) used a smartphone to complete the recall. So far, smartphone-based tools mainly reflected digital counterparts of FR that enable real-time recording. In contrast, in 24HDRs, the diet is recalled from the previous day. To take into account the ongoing digitalization, some web-based 24HDRs have already been optimized for mobile applications [28,35]. Although this further eases the administration for the user by giving him even more flexibility for conducting the 24HDR, it might also bear the risk that the boundaries between the dietary assessment methods become indistinct. Participants might mix up their diet from the current and past days.

The most challenging part in the adaptation process of myfood24-Germany was the development of an appropriate food database. The underlying database determines the users’ success of searching for food items and is essential for the functionality and accuracy of automated web-based dietary assessment tools [11,36]. Different approaches for database development were chosen for automated self-administered 24HDRs [10]. For myfood24-UK, an extensive database including generic and branded items (*n* = 40,274) was developed in an elaborate process [11]. For Foodbook24, a contrary approach was chosen by limiting the items in the underlying database to 751 generic food items [37]. The authors assumed that the limitation of food items limits participants’ burden and observed only small differences in estimated nutrient intake when comparing this concise food list with an extensive one (*n* = 2319 items). Database maintenance seems more feasible for a concise variant with generic items only, since the inclusion of branded products in the database involves the need for regular updates. However, data on user-experience to assess the actual level of burden associated with a lower versus a higher range of food choices in self-administered 24HDRs are missing. The myfood24-Germany database comprises a large number of generic items and branded products. Although most participants agreed that the search function in myfood24-Germany was easy to use, many reported having problems with finding a correct item and criticized the incompleteness of the database. This has also been observed for other web-based 24HDRs. For example, during the testing of myfood24-UK, 89% of the users stated that the search function was easy to use but only 29% agreed that they could find food items easily [5]. Rowland et al. identified finding food items as the most reported challenge by users of INTAKE24 during field-testing [31]. Notably, INTAKE24 offers a much smaller number of food items to choose from [10] than myfood24-UK and includes mainly generic items [28]. In the free text comments of the evaluation questionnaire, users of myfood24-Germany often recommended the addition of basic generic items as well as branded food products to the underlying database. Further, participants recommended changing the order of search results, to add more synonyms and (miss)spellings, and reduce the number of search results. This feedback may suggest that participants’ burden might be reduced by optimizing the search mechanisms rather than by minimizing the number of included food items in the underlying database. This hypothesis is supported by results from a comprehensive usability test of ASA24, where the improvement of the search functionality was named as one major key to solving usability issues [36]. Some participants drew a comparison to commercial food tracking systems and their more comprehensive food databases in the evaluation questionnaires. These crowd-sourced databases include a large number of branded products, suggesting that users do not struggle with the wide range of food choices and may prefer entering the exact product they consumed instead of searching for a generic equivalent. However, users of a commercial food logging app also had trouble finding correct food items in the database [38].

Our examination of users’ search strategy in myfood24-Germany (study 2) showed that branded products were mostly searched for by entering a generic description, suggesting that the products’ brand plays a minor role when searching for food items. Further, participants preferred to choose a related generic item as an equivalent for an absent branded product. Even when an exact match for a branded product was available in the database, a related generic item was chosen in nearly half of the cases. However, this behavior might be explained by the fact that generic items are displayed first in the search results. Users of online 24HDRs tend to choose items from the top of the list when several search results are displayed [39] and our evaluation study showed that the search filters for food groups or brands were rarely used. Further, the branded products presented in the sample meals were selected in the way that the majority were not specifically available in the database to force participants to search for alternative items. The lack of success in the search for branded products might have caused an adaptation in the search strategy. This is also supported by the fact that search terms included more brand names at the beginning of the entering process than at the end.

The comparison of hypothetical energy and nutrient intake showed that the difference between mean energy and macronutrient intake for the entered sample meals and the reference was low. However, the interindividual variation was high. The variation in hypothetical energy intakes primarily resulted from the different alternatives that were chosen for absent manufactured products. For example, for a branded smoothie product, two alternatives were chosen that differed around 150% in energy content. These findings support the presumption that searching and choosing an appropriate equivalent for products is a major challenge for participants. This is supported by results from ASA24, showing that participants substitute items they cannot find by potentially inappropriate other items or omit them [36].

Our study had some strengths and limitations. The study 1 design allowed a detailed evaluation of myfood24-Germany after repeated use by adult participants across a large age range in a study setting. The number of participants that answered an evaluation questionnaire exceeded the minimum number of five users necessary to identify usability problems within every age group [40]. Due to convenience sampling, the study population was not representative of the general German population. The majority of participants were female, highly educated, and familiar with using computers. Further, the examined sample included nutritionists and people who had had experience in completing a 24HDR. This limits the generalizability of our results. However, SUS score and time for completion did not differ after the exclusion of nutritionists and participants with experience in completing 24HDRs. Nevertheless, our study sample might include a number of participants with a high interest in nutrition or self-tracking, which could partly explain the willingness of participants to conduct repeated administrations of myfood24-Germany. Usability evaluation might have been more positive in the examined sample because the functionality of myfood24-Germany has been explained personally to all participants at first usage. To our knowledge, the study on users’ search behavior (study 2) is one of the first studies to give important insights on how participants work with free text search functions, and how equivalents for missing food items are selected. However, we only included a small number of participants, which limits the generalizability of our findings. Participants were asked to enter food items from a sample meal, which probably included foods that are not regularly consumed by participants. This might explain some of the difficulties users experienced when searching for an appropriate alternative.

## 5. Conclusions

Myfood24 was successfully adapted for use in the German adult population. To our knowledge, it is now the first fully automated online system for self-administered 24HDRs available in Germany. The user evaluation in the examined sample of adults indicated a short completion time, good usability and acceptability of the tool across age groups, and confirmed its feasibility for repeated short-term application. Search mechanisms should be optimized to facilitate the successful search for specific food items in the underlying database and decrease the potential risk of misreporting.

## Figures and Tables

**Table 1:** Characteristics of study participants who completed an evaluation questionnaire on myfood24-Germany (*n* = 92).

	Male Median (Q1–Q3)	Female Median (Q1–Q3)
Participants, *n* (%)	22 (24)	70 (76)
Age, years	27.5 (24.0–54.0)	50.0 (25.0–62.0)
BMI, kg/m^2^	23.1(20.8–24.4)	21.8 (19.6–23.7)
Smoker, *n* (%)	2 (9.1)	6 (8.6)
Education level, *n* (%)		
No school-leaving qualification	-	1 (1.4)
Trainee/Student	5 (22.7)	8 (11.4)
Vocational education	2 (9.1)	22 (31.4)
College/University degree	15 (68.2)	39 (55.7)
Type of diet, *n* (%)		
Omnivores	20 (90.9)	57 (81.4)
Vegetarians	1 (4.6)	9 (12.9)
Vegans	1 (4.6)	4 (5.7)
Nutrition professionals, *n* (%)	2 (9.1)	17 (24.3)
Conducted a 24HDR before, *n* (%)	-	10 (14.3)
Experience in using a computer/tablet, *n* (%)	19 (86.4)	52 (74.3)

Lower- (Q1) and upper-quartile (Q3).

**Table 2:** System usability scale (SUS) scores and calculated completion time for a 24HDR with myfood24-Germany stratified by age groups and for the total sample.

	Total Sample (17–78 Years)	Young Adults (≤30 Years)	Adults (31–65 Years)	Older Adults (>65 Years)
Participants, *n* (%)	92	46 (50)	32 (35)	14 (15)
Number of 24HDRs ^a^	367	183	128	56
Female sex, %	76	65	91	79
SUS score, median (Q1–Q3)	78 (66–84)	78 (65–85)	78 (66–85)	74 (70–83)
Completion time in minutes, median (Q1–Q3)	15 (9–23)	11 (7–16)	19 (13–28)	23 (16–32)

24HDR = 24-h dietary recall, lower-(Q1) and upper-quartile (Q3); ^a^ all participants conducted four 24HDRs with myfood24-Germany, except for one participant aged ≤30 years who conducted only three 24HDRS.

**Table 3:** Self-reported application and evaluation of available features in myfood24-Germany by participants completing an evaluation questionnaire (*n* = 92).

Feature in Myfood24	Used by *n* (%) of Participants	Considered Helpful by *n* (%) of Users
Optional quick list	35 (38)	31 (89)
Recipe builder	33 (36)	27 (82)
List of recently used items	9 (10)	8 (89)
Help function	6 (7)	5 (83)
Search filters	47 (51)	39 (83)

**Table 4:** Demographic characteristics of study participants who entered three sample meals into myfood24-Germany (*n* = 15).

	Total	Analysis 1	Analysis 2
**Participants, *n* (% female)**	15 (33)	14 (36)	9 (44)
Age, years ^a^	25 (21–39)	25 (21–32)	25 (21–32)
Educational status, *n* (%)			
High school graduation	3 (20)	3 (21)	2 (22)
Vocational education	3 (20)	3 (21)	2 (22)
College/university degree	9 (60)	8 (58)	5 (56)

^a^ median (Q1–Q3).

**Table 5:** Hypothetical energy and nutrient intake of the sample protocol and corresponding reference values (*n* = 9).

Nutrient	Sample Meal		Reference Values ^a^	Difference ^b^
	Mean	SD		Total (%)
Energy, kcal	2970	225	2928	42 (1.4)
Protein, g	83	4	81	2 (2.5)
Fat, g	135	16	131	4 (3.1)
Carbohydrates, g	345	22	316	29 (9.2)

SD = standard deviation; ^a^ reference values were determined by nutritional information on food packaging and the German Food Code and Nutrient Data Base (BLS); ^b^ difference between the mean values of *n* = 9 analyzed protocols and reference.

## Supplementary Materials

The following are available online at https://www.mdpi.com/2072-6643/12/1/160/s1, Figure S1: Flowchart of study participants and entered 24HDR eligible for analyses.
