# Supplementary material for: Adaptation and Evaluation of Myfood24-Germany: A Web-Based Self-Administered 24-h Dietary Recall for the German Adult Population
Source: Nutrients. 2020 Jan 6;12(1):160. doi: 10.3390/nu12010160 (PMC7019560; doi:10.3390/nu12010160)
Supplement: Supplementary file 1 [file nutrients-12-00160-s001.pdf]

Electronic Supplementary Material

Article in Nutriens

Adaptation and Evaluation of Myfood24-Germany: A Web-Based Self-Administered 24-h Dietary Recall for the German Adult Population

Stefanie A.J. Koch [1,*,†], Johanna Conrad [1,†], Linda Hierath [1], Neil Hancock [2], Sarah Beer [3], Janet E. Cade [2] and Ute Nöthlings [1]

[1] Nutritional Epidemiology, Department of Nutrition and Food Sciences, University of Bonn, 53115 Bonn, North Rhine-Westphalia, Germany; jconrad@uni-bonn.de (J.C.); lhierath@uni-bonn.de (L.H.); noethlings@uni-bonn.de (U.N.)

[2] Nutritional Epidemiology Group, School of Food Science and Nutrition, University of Leeds, Leeds LS2 9JT, UK; n.hancock@leeds.ac.uk (N.H.); J.E.Cade@leeds.ac.uk (J.E.C.)

[3] Dietary Assessment Ltd., Nexus, University of Leeds, Leeds LS2 3AA, UK; S.L.Beer@leeds.ac.uk

* Correspondence: s.koch@uni-bonn.de; Tel.: +49-228-736-9861

† These authors contributed equally to this work.

Electronic Supplementary Material

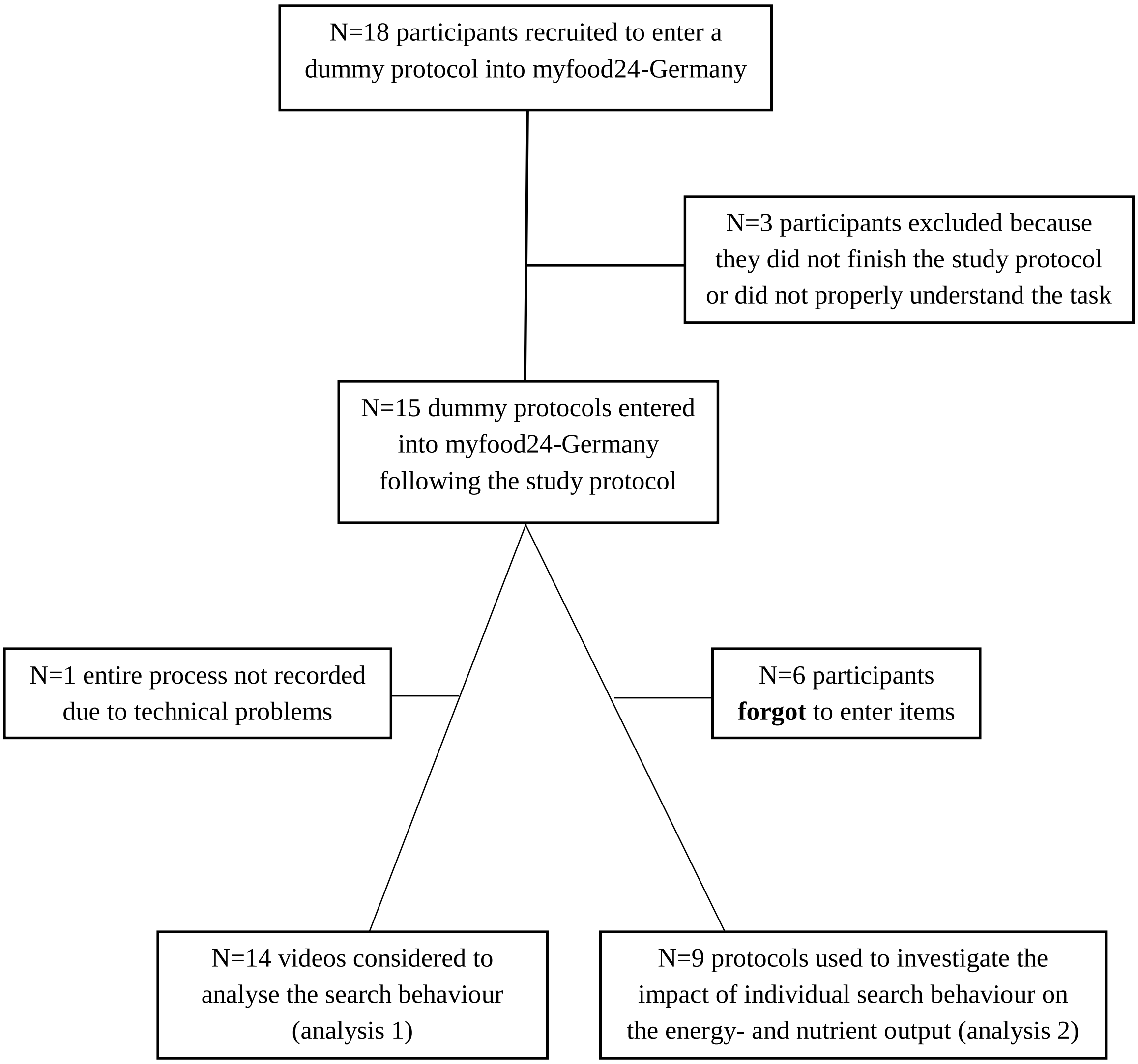

Figure S1: Flowchart of study participants and entered 24HDR eligible for analyses
